# Genetic Polymorphisms of Invasive *Ambrosia artemisiifolia* L. in Localities of Slovakia Accessed by Bet v 1 Homologs Differ in Discrimination of Accessions and Show Their Outcrossing in This Area

**DOI:** 10.3390/plants14172790

**Published:** 2025-09-05

**Authors:** Lucia Klongová, Adam Kováčik, Veronika Štefúnová, Monika Tóthová, Jana Žiarovská

**Affiliations:** 1 Department of Agrobiology, Research Centre of AgroBioTech, Slovak University of Agriculture in Nitra, 949 76 Nitra, Slovakia; 2 Institute of Plant and Environmental Sciences, Faculty of Agrobiology and Food Resources, Slovak University of Agriculture in Nitra, 949 76 Nitra, Slovakia; adam.kovacik@uniag.sk (A.K.); veronika.stefunova@uniag.sk (V.Š.); 3 Institute of Agrochemistry and Soil Science, Faculty of Agrobiology and Food Resources, Slovak University of Agriculture in Nitra, 949 76 Nitra, Slovakia; monika.tothova@uniag.sk

**Keywords:** common ragweed, allergen homologs, polymorphism

## Abstract

*Ambrosia artemisiifolia* is one of the invasive plants found in Europe. Research into this species is important not only in terms of environmental consequences but also in terms of human health, as it is an allergenic weed. Here, the genetic variability in the accessions of common ragweed from three localities of the Slovak Republic was analysed. A Bet v 1-based amplified polymorphism was applied for degenerated and non-degenerated primers. Three of the five primer variants were able to distinguish all analysed accessions, and two primer combinations were able to distinguish two accessions in the same amplified fingerprint profile from Kicsina. Using the BBAP technique, between of 1 to 16 amplicons were obtained per 1 genotype of common ragweed within a range of 68 to 3266 base pairs. Using two of the BBAP primer variants, unique fragments were obtained. The results indicate that the outcrossing among Slovak genotypes of *Ambrosia artemisiifolia* L. as non-specific BBAP profiles of individual analysed localities was observed.

## 1. Introduction

The genus *Ambrosia* consists of 46 botanical species, of which only *Ambrosia artemisiifolia* L. and *Ambrosia trifida* L. have been found growing in the Slovak Republic [1,2]. *A. trifida* occurs only randomly and not expansively, whereas *A. artemisiifolia* is commonly distributed, especially in the southern parts of the country [3]. A warm continental climate, dry soil, and adequate humidity during the summer are all necessary for Ambrosia species [4]. Typically, this species can be found on waste grounds, fallow lands, roadsides, railway tracks, and dry fields and pastures [5]. Common ragweed (*Ambrosia artemisiifolia* L.) is native to North America and considered an invasive plant in most European countries, including Slovakia [6,7].

In addition to being a health risk to humans, biological invasions pose a serious threat to the preservation of biological diversity and ecological services [8]. Because of its tremendous potential for expansion throughout Europe, common ragweed is regarded as a bioinvader [9]. It grows quickly and easily, mostly because each plant produces between 3000 and 62,000 seeds annually [10]. Furthermore, if the growth conditions are not optimal for ragweed seeds to germinate, they can lie dormant in the soil for over 30 years [11]. Because there is no efficient, natural method for ragweed seeds to disperse over long distances, human activity is primarily responsible for long-range ragweed dissemination [12]. Highways, railroads, soil transportation, and agricultural machinery are the most significant introduction channels associated with human activity [13]. Nevertheless, considering the regional scale, seeds can disperse on their own through wind, water, and birds [14,15]. Ragweed spreads spontaneously at a rate of 6–20 km annually [16]. Although *A. artemisiifolia* was first introduced to Europe in the second part of the 19th century, its widespread distribution started at the start of the 20th century following World War I [17,18]. Shipments of agricultural goods, such as purple clover seeds, potatoes, and maize, brought the species from America to Europe. The first documented temporary colonisation of this species in Europe occurred in Germany and France at the same time in 1863, according to herbarium records [19,20].

In addition to being a significant pest in agriculture and a source of pollen that causes allergies, ragweed is regarded as a noxious invading species [21]. In many parts of the United States [22] and Canada [23], its pollen is one of the most significant causes of allergy diseases, including conjunctivitis, asthma, seasonal rhinitis, and less frequently dermatitis. Even in Europe, its clinical significance has grown significantly in recent decades [24]. Sensitisation to ragweed pollen grains varies from 2.5% in Finland to 60% in Hungary among individuals with pollinosis in Europe [25]. Since a significant portion of pollen-allergic people respond to ragweed pollen grains, ragweed pollen is acknowledged as being extremely allergenic in Slovakia as well [26]. Additionally, the likelihood of sensitisation by *A. artemisiifolia* may be increased if there is cross-reactivity between the pollen allergens of various Asteraceae species [27]. Only a few pollen grains are sufficient to trigger allergy reactions in humans, while this anemophilous plant species generates millions of tiny pollen grains [28]. Each country has a different threshold value that causes clinical symptoms. The threshold value is less than 20 pollen grains m^−3^ in Austria [18,29], while it is less than 13 and 30 pollen grains m^−3^ in France and Hungary [19,30].

Ragweed’s pollen grains induce allergic respiratory disorders (pollinosis) in susceptible people, with common symptoms including rhinoconjunctivitis and asthma, in addition to their detrimental impact on biodiversity and crop production [31]. The primary pollination periods are thought to be late summer and fall [32], and it is projected that nearly 16 million persons in Europe are sensitised to ragweed pollen [4]. Amb a 1 is one of the main allergens found in ragweed pollen [33,34,35], among other allergenic molecules (Allergen nomenclature; http://www.allergen.org/; accessed 17 June 2025). Amb a 1 and Bet v 1 (the main birch pollen allergen) are both members of the larger PR-10 protein family, sharing significant sequence similarity and structural features, which is the basis of existing cross-reactivity. Strong cross-reactivity is seen with ragweed and mugwort, marshelder, and cocklebur [36]. The similarity between the genetic variability in PR-10 allergen sequences is well studied across various plant species [37,38] and has resulted in their use as DNA markers [39]. Here, a Bet v 1-based amplicon polymorphism (BBAP) [40] was used for the analysis of *A. artemisiifolia* for the first time. These markers are universal in their use in plants, as they are mapped from the homologs of allergen-coding genes and have been used to study Bet v 1 polymorphism variability among various plants [41].

In reality, no specific DNA marker-based information about the genetic diversity of invasive genotypes of common ragweed in Slovakia is available. Upon observing the closely distant localities of its distribution, it can be supposed that to be wind-pollinated, *A. artemisiifolia* outcrosses in Slovakia, and a gene-specific marker technique such as BBAP will show this. The objective of this study was to analyse the existing polymorphism among accessions from three localities using markers of Bet v 1 homologs and the BBAP technique.

## 2. Results

A set of non-degenerated forward primers as well as their degenerated variant [38] in the BBAP analysis of variability of *A. artemisiifolia* accessions from three localities of Slovakia were used. In all of the returned results, a basic amplicon with the length of 388 bp was obtained that was confirmed empirically as one of the checking amplicons for the BBAP technique, together with a set of other expected amplicons such as a 217 bp long amplicon for BBAP reverse R1 and a 550 bp long amplicon for BBAP reverse R3 [41].

### 2.1. BBAP Fingerprint Diversity Using the Degenerated Forward Primer

The degenerate primer pair amplified a total of 284 consistent amplicons distributed within 57 different size levels, of which all were polymorphic. No unique fragments were obtained. Fragment sizes ranged from 68 bp up to 2845 bp. A range of 2 up to 13 amplicons in individual common ragweed accessions was obtained.

**Figure 1:**
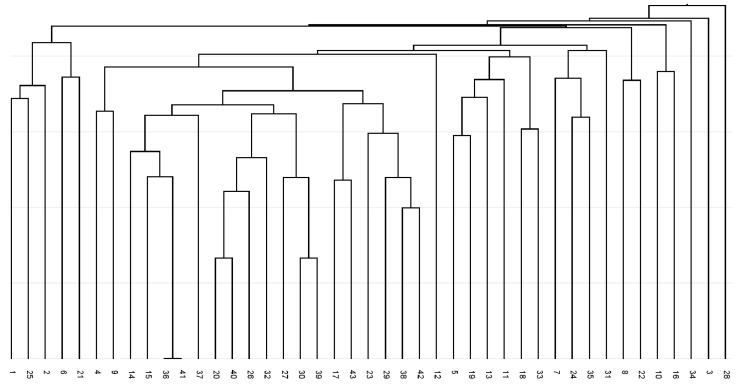
UPGMA dendrogram of Jaccard coefficients of similarity for analysed *Ambrosia artemisiifolia* for degenerate forward primer Bet v 1-based amplicon polymorphism fingerprints.

The constructed dendrogram (Figure 1) showed the separation of fingerprints profiles except for two genotypes from the Kiscina locality (36 and 41). A total of seven branches were generated in the dendrogram and no locality-specific grouping of any analysed accessions can be seen. Three genotypes, namely 3, 28, and 34, revealed the most distinctive BBAP/degenerate forward primer fingerprint profiles.

**Figure 2:**
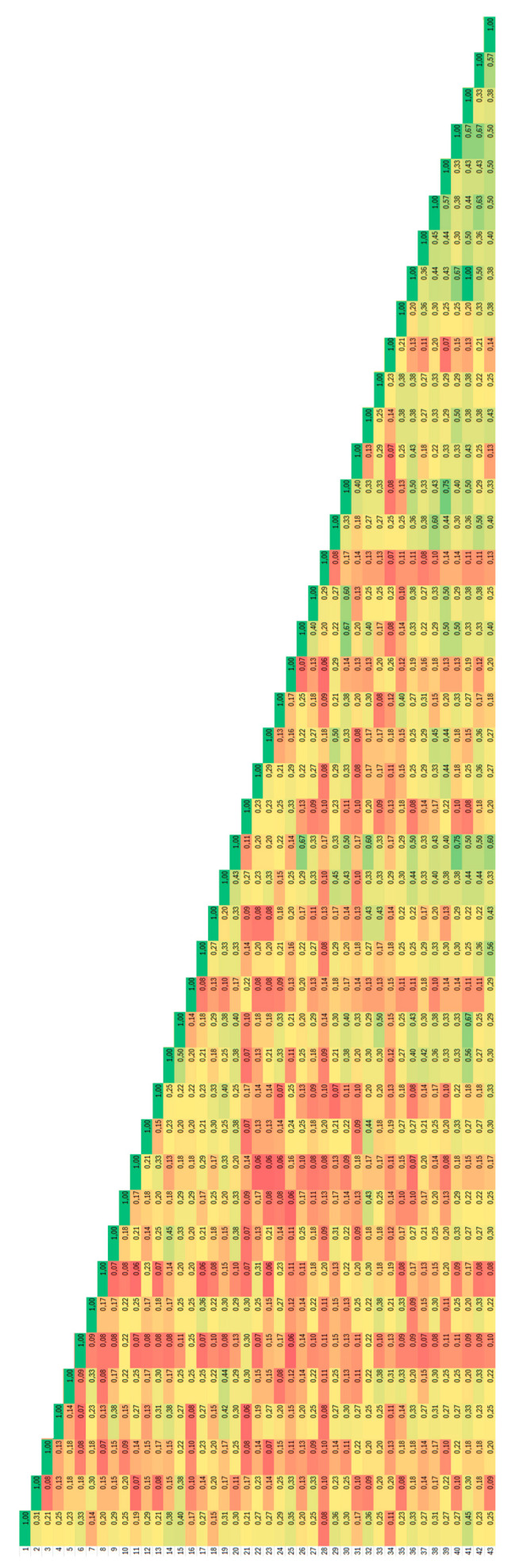
Heatmap distribution of values of Jaccard coefficients of similarity for *Ambrosia artemisiifolia* accessions analysed by degenerate BBAP. Dark red colour corresponds to 0.00 and dark green colour to 1.0.

The most similar genotypes were those from the Kicsina locality (Figure 2), where the Jaccard coefficient of genetic similarity has an average of 0.43, and the accessions with the same fingerprint were from this locality too.

### 2.2. BBAP Fingerprint Diversity Using the Non-Degenerated Variants of Forward Primer

A total of 416 consistent amplicons, which were distributed within 53 different size levels, of which all were polymorphic, were amplified in the case of the F1 (5′aaccacaccatcaccgac3′) primer pair using BBAP analysis. No unique fragments were obtained. Fragment sizes ranged from 69 bp up to 1232 bp. A range of 3 up to 15 amplicons in individual common ragweed accessions was obtained.

**Figure 3:**
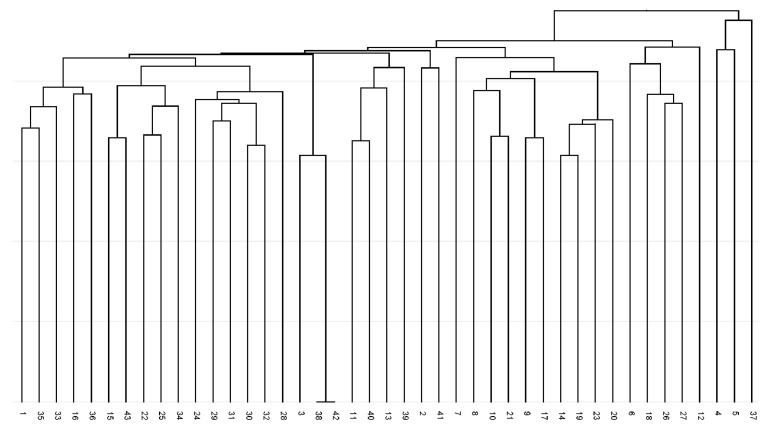
UPGMA dendrogram of Jaccard coefficients of similarity for analysed *Ambrosia artemisiifolia* for F1 non-degenerate forward primer Bet v 1-based amplicon polymorphism fingerprints.

The constructed dendrogram (Figure 3) showed the separation of fingerprint profiles except for two genotypes from the Kiscina locality (38 and 42). A total of nine branches were separated in the dendrogram, and no locality-specific grouping of analysed accessions was obtained.

**Figure 4:**
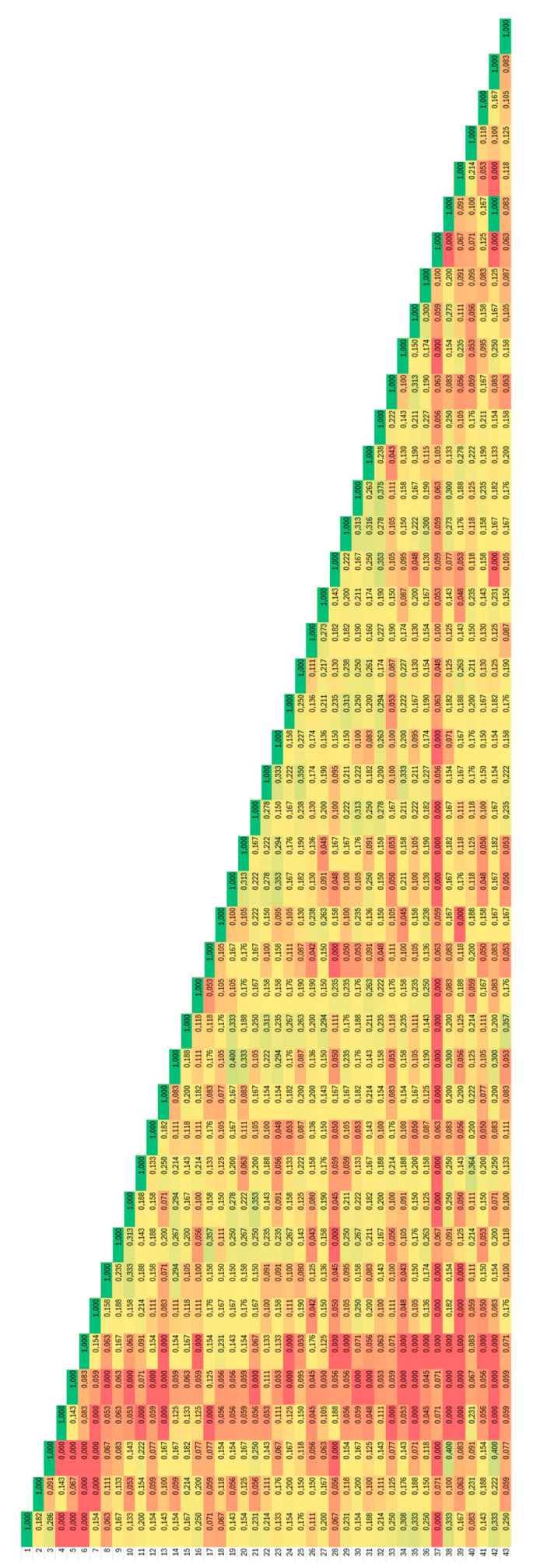
Heatmap distribution of values of Jaccard coefficients of similarity for *Ambrosia artemisiifolia* accessions analysed by F1 non-degenerate BBAP. Dark red colour corresponds to 0.00 and dark green colour to 1.0.

In this primer combination, the accessions shared only low genetic similarity with the average value of the Jaccard coefficient of 0.159, and among some samples from the Balvany locality, completely different fingerprints have been obtained (Figure 4).

In the case of the F2 (5′aaccacaccatcaacgac3′) primer pair used in BBAP analysis, a total of 442 consistent amplicons were amplified that were distributed within 62 different size levels, of which all were polymorphic. No unique fragments were obtained. Fragment sizes ranged from 68 bp up to 3266 bp. A range of 5 up to 16 amplicons in individual common ragweed accessions was obtained.

**Figure 5:**
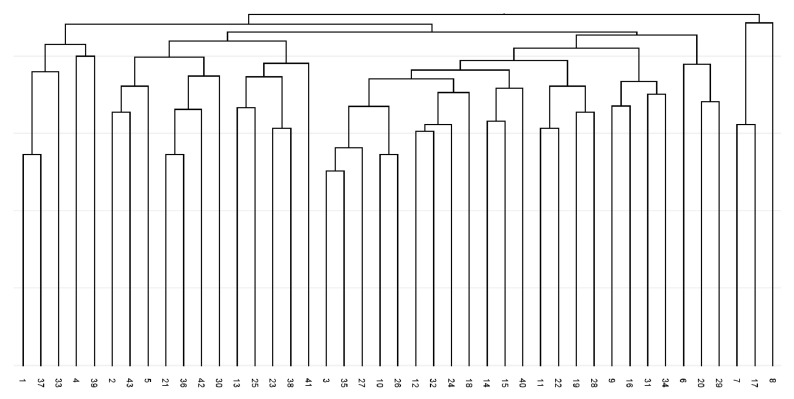
UPGMA dendrogram of Jaccard coefficients of similarity for analysed *Ambrosia artemisiifolia* for F2 non-degenerate forward primer Bet v 1-based amplicon polymorphism fingerprints.

The constructed dendrogram (Figure 5) showed the separation of all analysed accessions from all three localities. A total of ten branches were obtained in the dendrogram, and no locality-specific grouping of analysed accessions exists. Three genotypes from the Balvany locality (7, 8, and 17) with different profiles from all other analysed accessions were obtained.

**Figure 6:**
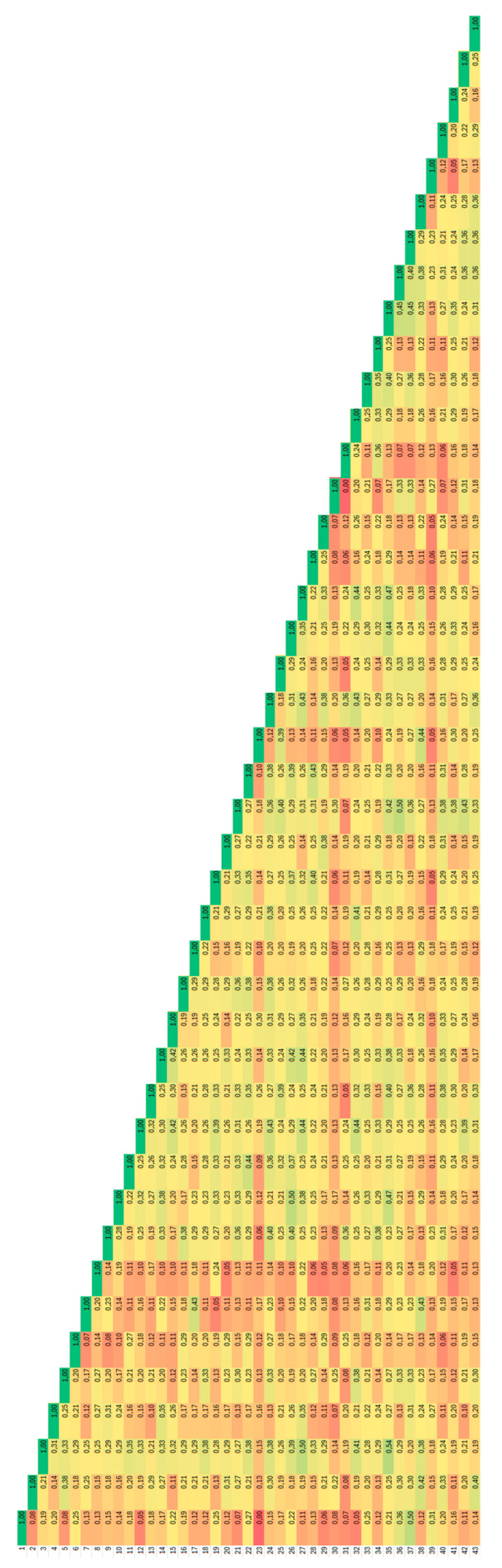
Heatmap distribution of values of Jaccard coefficients of similarity for *Ambrosia artemisiifolia* accessions analysed by F2 non-degenerate BBAP. Dark red colour corresponds to 0.00 and dark green colour to 1.0.

In the F2 non-degenerate primer combination, the accessions shared the narrowest genetic similarity with the average value of the Jaccard coefficient being 0.24, and among the samples from Balvany (9–19), very similar fingerprints were obtained (Figure 6).

A total of 379 consistent amplicons, which were distributed within 55 different size levels, of which all were polymorphic, were amplified in the case of the F3 (5′aaccacaccatgaccgac3′) primer pair using BBAP analysis. Here, two unique fragments in accession 9, with the length of 2256 bp, and in accession 5, with 1701 bp, were obtained. Fragment sizes ranged from 68 bp up to 2256 bp. A range of 5 to 13 amplicons was obtained in individual common ragweed accessions. Here, no amplification was seen in accessions 17, 22, 24, and 35.

**Figure 7:**
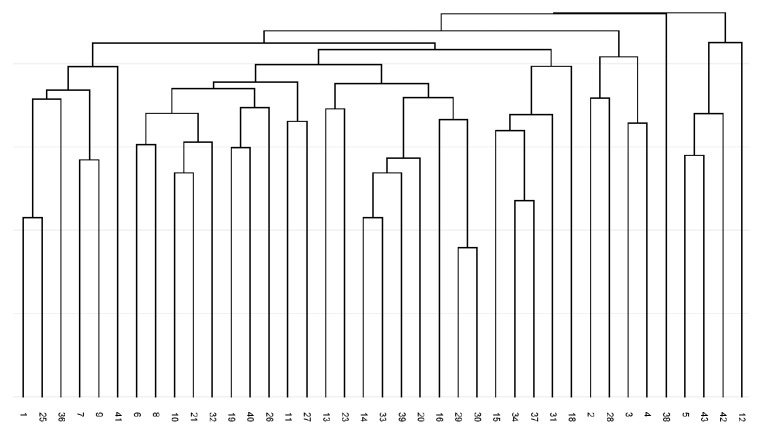
UPGMA dendrogram of Jaccard coefficients of similarity for analysed *Ambrosia artemisiifolia* for F3 non-degenerate forward primer Bet v 1-based amplicon polymorphism fingerprints.

The constructed dendrogram (Figure 7) shows the separation of all analysed accessions from all three localities. A total of six branches are in the dendrogram, with no locality-specific grouping of analysed accessions.

**Figure 8:**
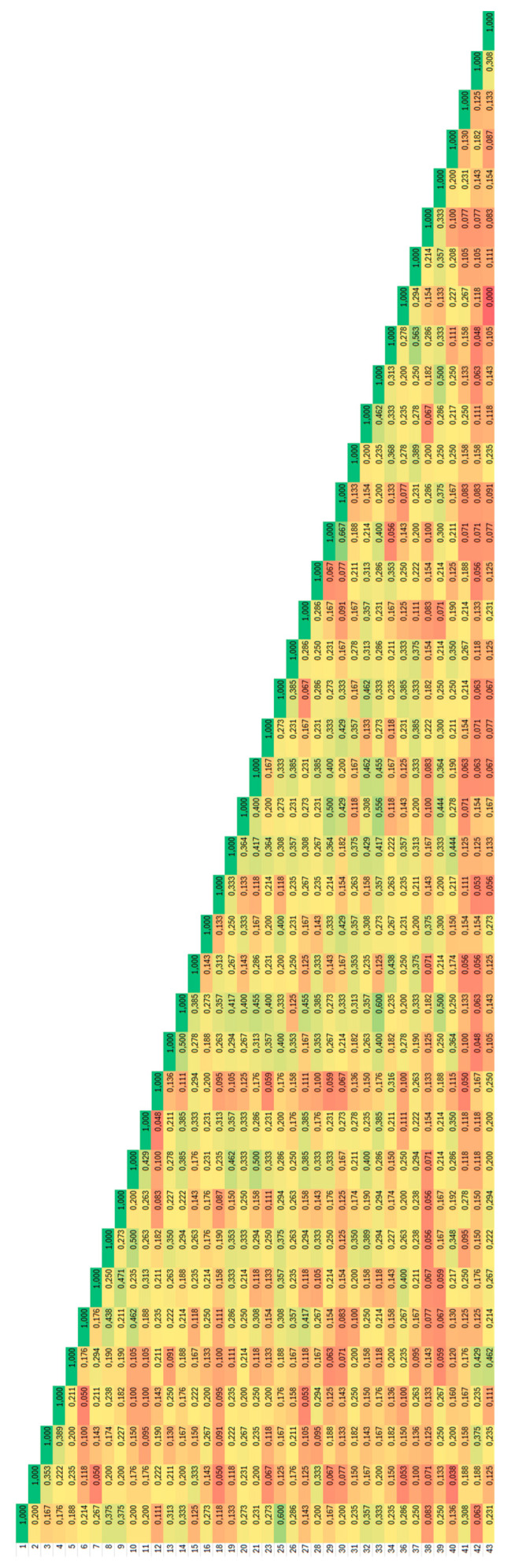
Heatmap distribution of values of Jaccard coefficients of similarity for *Ambrosia artemisiifolia* accessions analysed by F3 non-degenerate BBAP. Dark red colour corresponds to 0.00 and dark green colour to 1.0.

In the F3 non-degenerate primer combination, the accessions shared variable genetic similarity with the average value of the Jaccard coefficient being 0.238; the lowest was among the samples from Kiscina (Figure 8).

Only a limited amplification was obtained for common ragweed accessions with only a total of 170 amplicons. But here, different unique fragments were obtained in accession 28 with the length of 365 bp; 12 with the length of 529 bp; 21 with the length of 612 bp; 9 with lengths of 676 bp, 880 bp, and 1253 bp; 16 with the length of 698 bp; 33 with the length of 746 bp; and in accession 13 with lengths of 1422 bp, 1589 bp, 1967 bp, and 2656 bp in the case of the F4 (5′aaccacaccatgaacgac3′) primer pair using BBAP analysis. Fragment sizes ranged from 87 bp up to 2656 bp. A range of 5 to 13 amplicons was obtained in individual common ragweed accessions. Here, no amplification was seen in genotypes 17, 22, and 24.

**Figure 9:**
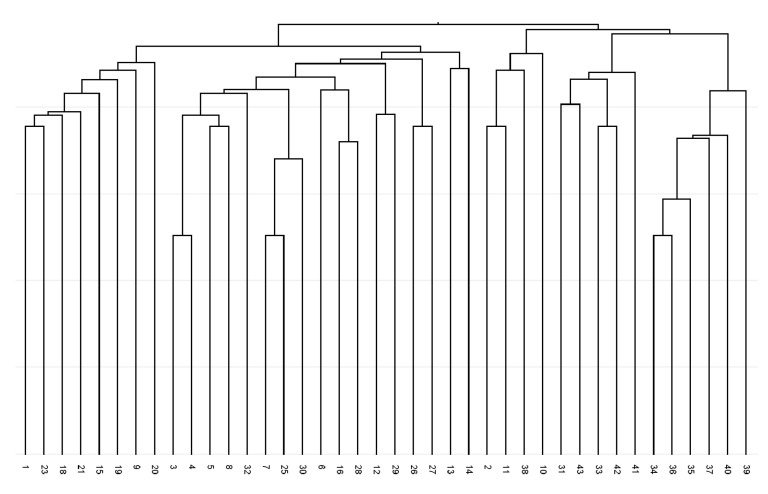
UPGMA dendrogram of Jaccard coefficients of similarity for analysed *Ambrosia artemisiifolia* for F4 non-degenerate forward primer Bet v 1-based amplicon polymorphism fingerprints.

The constructed dendrogram (Figure 9) shows the separation of all analysed accessions from all three localities. A total of five branches were obtained, and a separate branch of most of the accessions from the Kicina locality was obtained (except for accessions 30 and 32).

**Figure 10:**
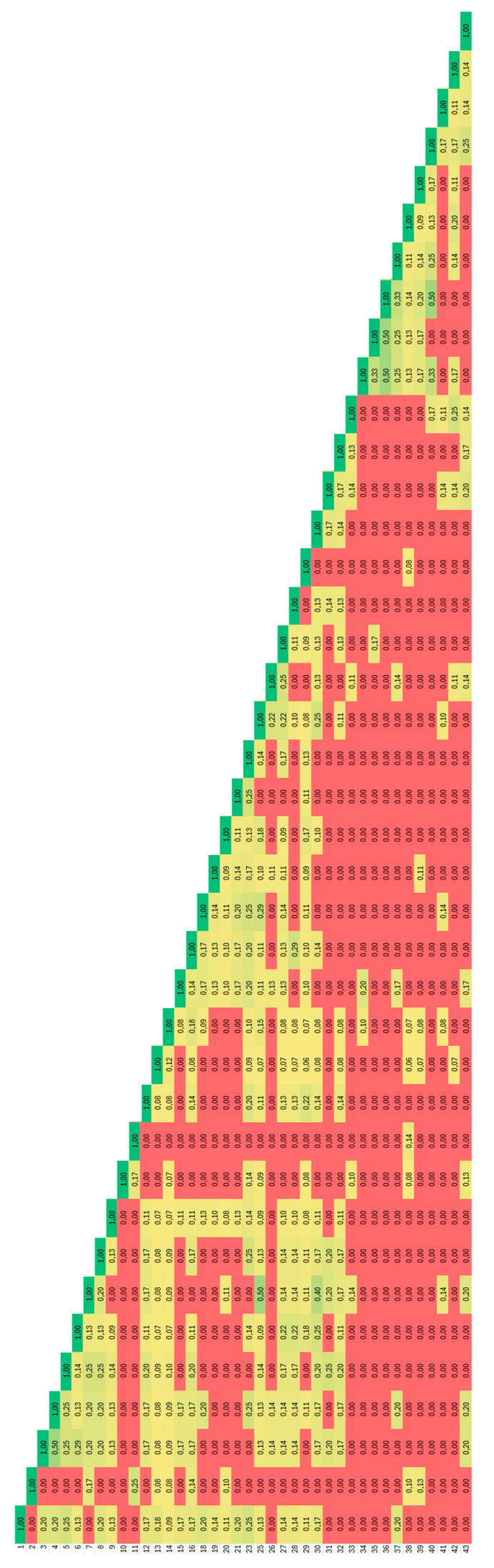
Heatmap distribution of values of Jaccard coefficients of similarity for *Ambrosia artemisiifolia* accessions analysed by F4 non-degenerate BBAP. Dark red colour corresponds to 0.00 and dark green colour to 1.0.

In the F4 non-degenerate primer combination, the accessions were quite distinctive in their paired genetic similarity regarding the Jaccard coefficient (Figure 10).

### 2.3. Comparison of Obtained BBAP Fingerprint Profiles for Individual Primer Combinations

Comparing the individual types of degenerated/non-degenerated forward primers used in the study, the F3 non-degenerated forward primer in BBAP was the most effective in distinguishing the analysed common ragweed (Table 1). For degenerated and F1 non-degenerated primers, two of the analysed accessions provided the same fingerprinting pattern. These primer variants were not able to distinguish all of the analysed common ragweed accessions. In the case of the F2 non-degenerated forward primer in BBAP, 90% of the analysed common ragweed accessions had Jaccard similarity indices lower than 0.5, and the discrimination power of this technique was lower when compared to the other techniques used here. The ability of individual primer variants of BBAP to detect polymorphisms was comparable for variants F degenerate/F1 non-degenerate/F2 non-degenerate/F3 non-degenerate, with an average PIC value of 0.266. The lowest effectiveness for the polymorphism analysis of *Ambrosia artemisiifolia* variability, as the marker index value was lowest among the five used combinations, was provided by primer variant F4 non-degenerate.

## 3. Discussion

The number of biological invasions has significantly expanded over the past few decades as a result of globalisation, and fresh introductions do not seem to be slowing down [42]. Invasive species can cost the economy a lot of money and have a variety of negative impacts on ecosystem services and biodiversity [43,44,45,46]. Originally from North America, common ragweed has spread around the world. More than 30 nations in Europe consider it invasive [47], and as the climate changes, its impact and spread are expected to rise [48,49,50]. In Europe, ambrosia-induced allergies affected 13.5 (95% CI 10.9–14.8) million people, resulting in yearly economic expenses of roughly EUR 7.4 billion [6].

The genetic diversity and population structure of *Ambrosia artemisiifolia* were revealed by different types of DNA markers, but BBAP-based variability is analysed here for the first time. Microsatellite studies in France found that invasive populations display genetic diversity levels comparable to/higher to those in North America. This contrasts with the native range, where within-population diversity was lower [51,52]. Historic herbarium-based comparisons revealed that recent French populations show greater diversity and less structure than early invasions because of extensive gene flow and admixture across introduced populations [53]. New genomic and expressed sequence tag SSR markers revealed that two major introductory events shaped European populations, followed by secondary spread. Genetic structure analyses suggest six genetic clusters within Europe and North America [52]. In Slovakia, two main starting points of common ragweed invasions were identified, situated in the southwest and southeast of the country, where the southwest is the older one. Based on the results of BBAP analysis introduced here, their profiles of Bet v 1-based polymorphism do not show distinctive fingerprints generated, which is what they pointed out to their outcrossing. This is in concordance with the knowledge that one of the most important factors for success of invasive plant species is their intraspecific hybridization, which substantially increases their adaptability to new habitats [54]. Experiments in French invasive populations found significant adaptive divergence of common ragweed, especially in reproductive allocation relative to neutral genetic variation. This indicates selection, likely in response to local abiotic gradients like altitude and latitude [55], and genome-wide resequencing of >600 modern and historic samples from North America and Europe revealed genomic regions under climate-mediated selection. Several large haploblocks, some likely due to chromosome inversions, are associated with climatic adaptations and increased frequency over time in Europe [56]. In Slovakia, nuclear DNA content varies modestly (2.08–2.27 pg/2C). However, links between genome size and environment remain uncertain [57].

BBAP was proved to be effective in the distinguishing of common ragweed genotypes. An in silico analysis of the amino acid sequences reported for the Bet v 1 allergen reveals a highly varied identity among plant species [58]. This offers the possibility of analysing the variability of these genes in plants by using conservative regions as DNA-based markers. The verified IgE epitope is included in the comparatively high homology of amino acid sequences in the forward primer region for the BBAP method [59]. The primary homologous Bet v 1 allergen that causes more than 95% of allergy reactions in people with sensitivities to common ragweed is Amb a 1, an acidic, non-glycosylated 38 kDa protein belonging to the pectate lyase family [60,61]. The pollen intine layer is where the Amb a 1 protein is primarily bound [62]. Based on the amount of the allergen in the pollen grain, the Pollen Allergen Potency (PAP) can be calculated. The precise mechanism underlying this variability is yet to be understood and the PAP value is not constant and varies throughout the year and between seasons and places depending on environmental factors [63].

Reverse primers of BBAP match the amino acid variability at position 119 of the Bet v 1 protein (P15494) and amplify a variable region of the year-10 gene of Bet v 1 [58]. The effectiveness of the in silico generated markers in providing polymorphic fingerprints among various plant accessions has been demonstrated up to now for different plant species. In previous studies, the intraspecific variability of *Malus domestica* Borkh. cultivars was analysed using degenerate primers that anneal a variable and conserved portion of PR-10 protein homologous genes in the BBAP approach [64]. The formation of amplicons and comparatively monomorphic profiles demonstrated the stability of the specified Bet v 1 isoforms within the chosen apple cultivars. In the analysis of different Lamiaceae species, the F2 and F3 primer pair combinations of BBAP showed the similarity of basil and oregano species. In the same study, F1 showed the similarities for basil with common sage and for rosemary with creeping thyme. F4 showed the similarity for basil with peppermint and oregano with common thyme [65]. The study of the length of polymorphism variability of the Bet v 1 homologue in orange varieties by using degenerated and non-degenerated primers provided the results where degenerated primer combinations and only one of the non-degenerated variants of primers generated fingerprints that were unique for every individual variety of analysed oranges. This indicates a higher degree of Bet v 1 homologue sequential conservativity when compared to other fruit species [66]. The universal applicability of BBAP indicates that the technique can be utilised across multiple vegetable and fruit species, allowing an efficient and reliable analysis of genetic variability. Identifying and understanding the distribution and genetic characteristics of alien plant species, such as Asteraceae, is a crucial prerequisite not only for native biodiversity conservation [67,68] but also for developing an effective strategy for their management.

## 4. Materials and Methods

### 4.1. Biological Material

**Figure 11:**
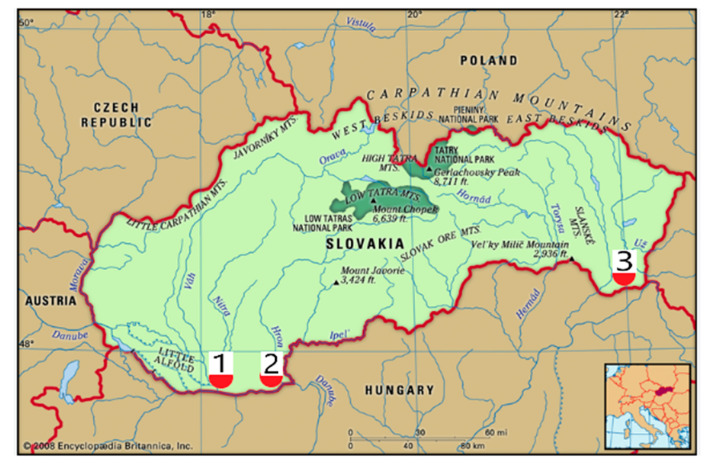
Localisation of localities of *Ambrosia artemisiifolia* collection. 1—Balvany; 2—Kiscina (Malá nad Hronom); 3—Veľký Horeš.

A total of 43 biological accessions of *Ambrosia artemisiifolia* L. were collected in 3 localities of southern parts of the Slovak Republic (Figure 11). All the accessions were transported into the laboratory immediately and kept frozen until further analysis. From the locality Balvany, accessions 1–20 were collected; from the locality Veľký Horeš, accessions 21–29 were collected; and from the locality Kiscina (Malá nad Hronom), accessions 30–43 were collected.

### 4.2. DNA Extraction

Total genomic DNA was extracted using the NucleoSpin^®^Plant II kit (Macherey-Nagel; Bratislava; Slovak Republic), following the instructions of the manufacturer. The quantity and quality of extracted DNA were checked using a NanoPhotometer^®^ P360 (Implen; Bratislava; Slovak Republic).

### 4.3. PCR Amplifications and Data Analysis

In this study, primers targeting Bet v 1 genes were used. Four primer pairs for the development of the BBAP technique for the comparison of triplets for different amino acids (histidine/asparagine/glutamine/lysine) were designed. In developing primers, the authors focused specifically on two amino acid segments. These segments were subjected to a BLAST 2.0.17.0 (Basic Local Alignment Search Tool) analysis with fruit species with established genomic sequences. The forward primer was designed for a region of high homology in *Malus domestica* [41]. Reverse primers were as follows: BBAP reverse R1/5′aaccacaccatcaccgac3′; BBAP reverse R2/5′aaccacaccatcaacgac3′; BBAP reverse R3/5′aaccacaccatgaccgac3′; BBAP reverse R4/5′aaccacaccatgaacgac3’. Every reverse primer has degeneracy situated at positions 12 (S) and 14 (K), meaning that position 12 can be occupied by either guanine or cytosine, and position 14 can be filled with either thymine or guanine [64,69].

PCR amplifications were performed using a TProfessional Basic Gradient XL thermocycler (BIOMETRA, Jena, Germany) in a total reaction volume of 10 µL, containing the following components: a combination of one forward primer with five reverse primers at a final concentration of 400 nM, 2 µL of genomic DNA diluted 1:500, EliZyme HS Robust MIX (Elisabeth Pharmacon), and PCR-grade water adjusted to the final volume. The thermal cycling conditions were as follows: initial denaturation at 95 °C for 5 min, followed by 40 cycles of denaturation at 95 °C for 45 s, primer annealing at 54 °C for 45 s, and extension at 72 °C for 35 s. A final extension was performed at 72 °C for 10 min, according to the optimised method of [70]. PCR products were separated by electrophoresis on 2% agarose gels (AGE) stained with GelRed^®^ Nucleic Acid Gel Stain (BIOTIUM, Fremont, CA, USA) and visualised using a UV transilluminator BDAdigital System 30 (Analytik Jena, Jena, Germany). Gel images were analysed with GelJ 2.0 software [71]. A binary matrix was generated based on the presence (1) or absence (0) of bands at specific sizes, and the resulting binary data were further analysed using the iMEC software [72] to calculate polymorphism indices.

## 5. Conclusions

Knowledge of Bet v 1 homologs is increasing, but the knowledge about their potential applicability in different genomic techniques is still limited. Here, the Bet v 1-based amplified polymorphic fingerprints for their applicability for the screening of Slovak common ragweed variability were analysed. Obtained results display an appropriate effectivity needed for distinguishing the genotypes of this species. Our findings indicate the outcrossing among Slovak genotypes of *Ambrosia artemisiifolia* L. is as a result of their widespread invasion and non-specific BBAP profiles of individual analysed localities. Understanding genetic diversity and linking it to DNA markers of this invasive plant will improve its control strategies in the future. Knowing genetic variation among invasive populations can help predict susceptibility to biological control agents. Revealing the knowledge about coding space and linking it to specific traits of common ragweed will provide possibilities in monitoring spread and population dynamics to elucidate introduction history and genetic diversity. Finding effective DNA markers to distinguish individual genotypes or ecotypes is important to develop rapid screening methods to track the changes in plant populations. That is why further research must continue.

## Figures and Tables

**Table 1:** Characteristics of fingerprints obtained by individual primer combinations of BBAP markers.

Forward Primer	PIC	AN	MI	D
F degenerate	0.184	6.6	0.00056	0.99
F1 non-degenerate	0.25	9.67	0.0013	0.97
F2 non-degenerate	0.26	10.28	0.299	0.24
F3 non-degenerate	0.37	8.81	0.41	0.38
F4 non-degenerate	0.16	3.95	0.0004	0.99

PIC—polymorphism information content; AN—average number of amplified alleles per accession; MI—marker index; D—discriminating power.

## Data Availability

The data presented in this study are available on request from the corresponding author.

## Abbreviations

The following abbreviations are used in this manuscript:

AN	Average number of amplified alleles per accession
Amb a 1	*Ambrosia artemisiifolia* 1 main pollen allergen
BBAP	Bet v 1-based amplified polymorphism
Bet v 1	*Betula verrucosa* 1 main pollen allergen
CI	Confidence interval
D	Discriminating power
DNA	Deoxyribonucleic acid
IgE	Immunoglobulin E
MI	Marker index
PCR	Polymerase chain reaction
PIC	Polymorphism information content
PR-10	Pathogen-related protein class 10
SSR	Single sequence repeats
UPGMA	Unweighted Pair Group Method with Arithmetic Mean
